# Protective effect of Haoqin Qingdan decoction on pulmonary and intestinal injury in mice with influenza viral pneumonia

**DOI:** 10.3389/fphar.2024.1449322

**Published:** 2024-12-06

**Authors:** Xi Lin, Jian Lin, Lichun Ji, Jiaona Zhang, Yezi Zhang, Junbin Hong, Geng Li, Xingdong Lin

**Affiliations:** ^1^ The Third Clinical Medical College, Guangzhou University of Chinese Medicine, Guangzhou, China; ^2^ Guangzhou University of Chinese Medicine, Guangzhou, China; ^3^ Chinese Medicine Guangdong Laboratory, Guangdong, China; ^4^ State Key Laboratory of Traditional Chinese Medicine Syndrome, Guangzhou University of Chinese Medicine, Guangzhou, China; ^5^ The Second Clinical Medical College, Guangzhou University of Chinese Medicine, Guangzhou, China; ^6^ Animal Experiment Center, Guangzhou University of Chinese Medicine, Guangzhou, China; ^7^ The Third Affiliated Hospital, Guangzhou University of Chinese Medicine, Guangzhou, China

**Keywords:** Haoqin Qingdan decoction, influenza viral pneumonia, network analysis, intestinal flora, traditional Chinese medicine

## Abstract

**Background:**

Haoqin Qingdan decoction (HQQD), composed of eleven herbs, is a traditional Chinese formula widely recognized for its efficacy in treating pulmonary inflammation induced by viral infections. Despite its extensive use, the potential pulmonary and intestinal protective effects of HQQD on influenza viral pneumonia (IVP) and the underlying molecular mechanisms remain unclear.

**Materials and Methods:**

Ultra-high-performance liquid chromatography coupled with mass spectrometry (UHPLC-MS) was employed to identify the major chemical constituents of the prescription. Subsequently, network analysis was conducted to predict the potential therapeutic targets of HQQD in IVP. The mechanisms by which HQQD mitigates lung and intestinal damage were further elucidated by assessing NP protein expression, inflammatory factors, TLR7/MyD88/NF-κB signaling pathway mRNAs and proteins, and through intestinal flora analysis.

**Results:**

The protective effects of HQQD on pulmonary and intestinal injuries induced by IVP were thoroughly investigated using comprehensive network analysis, signaling pathway validation, and gut microflora analysis. UHPLC-MS analysis identified the primary chemical constituents. Validation experiments demonstrated a significant reduction in NP protein expression in the lungs. HQQD notably alleviated immune damage in the lungs and intestines of mice by inhibiting NP protein expression and the release of inflammatory factors such as interleukin-6 (IL-6), interleukin-1β (IL-1β), tumor necrosis factor-alpha (TNF-α) and interferon-gamma (IFN-γ); downregulating the expression levels of TLR7, MyD88, and phospho-NF-κB p65 (p-p65); lowering serum LPS levels; and reducing the relative abundance of *Proteobacteria*.

**Conclusion:**

HQQD exerts therapeutic effects against influenza viral pneumonia through antiviral and anti-inflammatory mechanisms and by remodeling the intestinal flora. This study provides initial insights into the “gut-lung” axis mechanism of HQQD in combating respiratory influenza virus infection.

## 1 Introduction

Influenza A virus (IAV) is the primary driver of global influenza pandemics and poses significant risks to both the international economy and public health. According to the WHO, seasonal influenza viruses, including H1N1 and H3N2 subtypes of influenza A and influenza B viruses, are responsible for approximately 3–5 million severe cases and hundreds of thousands of deaths annually (Disease and Prevalence, 2016). Influenza viral pneumonia is a frequent complication of influenza infection and a major contributor to influenza-related mortality (Cavallazzi and Ramirez, 2018). The excessive release of inflammatory cytokines triggered by influenza infection can result in severe disease and life-threatening conditions (Ryabkova et al., 2021; Lobo et al., 2019). A hyperactivated immune response leads to the destruction of lung tissue, acute respiratory distress syndrome, and multiple organ failure (Kalil and Thomas, 2019). Despite the widespread availability of vaccines and antiviral therapies, their effectiveness is often compromised by antigenic drift and mutations in the virus, limiting their protective capabilities (Brody, 2019; Uyeki et al., 2019; Klomp et al., 2021; Batool et al., 2023). Modern antiviral treatments fail to fully prevent the disease progression associated with cytokine storms (Yao et al., 2022). Consequently, there is an urgent need to explore alternative therapeutic strategies for influenza viral pneumonia.

Recent studies have revealed the integrative nature of pulmonary and intestinal tissues, highlighting the interrelated immunological responses of the pulmonary and intestinal mucosa and the synchronous ecological changes between the lungs and intestines (Budden et al., 2017; Huang et al., 2018; Massart and Hunt, 2020). For instance, lipopolysaccharide (LPS) stimulation of the lungs in model mice has been shown to cause a significant increase in intestinal bacterial populations, underscoring the synchrony within the lung-gut axis (Sze et al., 2014). Beyond causing respiratory conditions like acute lung injury, influenza also impacts the digestive tract, altering the composition and structure of the gut microbiota (Zhang et al., 2020). Pulmonary infection-induced ischemia and hypoxia can indirectly damage the intestinal mucosal barrier, while intestinal bacterial imbalances due to barrier disruption may exacerbate lung injury by affecting both intestinal and pulmonary mucosal immunity (Dang and Marsland, 2019; de Oliveira et al., 2021). The gut microbiota’s influence on pulmonary immunity through the lung-gut axis has been well-documented (Taylor et al., 2016). This interconnectedness between lung and gut ecology illustrates a critical communication pathway between these organs. However, for viral infectious diseases, particularly respiratory influenza infections, it remains unexplored whether HQQD can alleviate symptoms, shorten treatment duration, and exert therapeutic effects *via* the lung-gut axis.

Traditional Chinese Medicine (TCM) exhibits unique efficacy in combating influenza by enhancing the body’s overall condition and mobilizing immune functions, thereby strengthening resistance to viral infections. This characteristic adaptability, distinct advantages, and broad development prospects make TCM particularly valuable. Haoqin Qingdan Decoction (HQQD), a traditional Chinese formula, is composed of Sweet Wormwood Herb, Baical Skullcap Root, Dried Tangerine Peel, Pinellia Tuber, Bamboo Shavings, Indian Buead, Seville Orange Fruit, Indigowoad Leaf, Indigowoad Root, Liquorice Root, and Talcum (Luo et al., 2016; Zhang et al., 2013). HQQD has been widely utilized in the treatment of viral infectious diseases (Yuan et al., 2020; Pan et al., 2010). The antiviral and anti-inflammatory properties of its components are closely linked to their underlying mechanisms of action. For instance, artesunate, an extract of *Artemisia annua L.*, has demonstrated potent suppression of IAV replication both *in vitro* and *in vivo*, while also inhibiting the release of proinflammatory cytokines such as TNF-α (Yang et al., 2023; Zhao et al., 2017). Dihydroartemisinin, the primary active metabolite of artemisinin, has been shown to mitigate the inflammatory cytokine storm by inhibiting the NF-κB signaling pathway (Huang et al., 2019). Additionally, oral administration of *A. annua L*. hot-water extract has potential as a cost-effective treatment for coronavirus disease 2019 (COVID-19) (Nair et al., 2022; Nair et al., 2023). *Scutellariae Radix* extract has shown considerable potential in treating acute lung injury induced by IAV (Zhi et al., 2019). Furthermore, *Isatidis Radix*-derived extract has been identified as a potential adjunctive antiviral therapy for IAV infections (Li et al., 2017; Nie et al., 2020). Animal experiments have confirmed the efficacy of HQQD in treating influenza viral pneumonia (IVP) (Luo et al., 2016; Zhang et al., 2013). However, the precise mechanisms underlying its pulmonary protective effects remain unclear, and it is yet to be determined whether HQQD’s therapeutic action against IVP involves modulation of the intestinal flora and reduction of intestinal injury.

TCM formulas typically exhibit multicomponent, multitarget synergistic effects (Luo et al., 2020; Yuan et al., 2017). Recently, network analysis has become a widely applied approach to investigate the mechanisms of TCM in treating complex diseases (Zhang et al., 2019). In this study, we aimed to predict the active compounds and potential targets of HQQD through network analysis, followed by experimental validation of the pathways involved in its efficacy against IVP. Our goal was to confirm the effectiveness of HQQD and elucidate the pulmonary-gut axis mechanism in resisting respiratory viral infections. This research seeks to clarify the mechanisms through which TCM formulas intervene in IVP and to provide a foundational basis for expanding clinical treatment options.

## 2 Materials and methods

### 2.1 Network construction and analysis

#### 2.1.1 Screening of HQQD candidate compounds

The canonical SMILES of each compound from UHPLC-MS analysis were collected from the PubChem database (https://pubchem.ncbi.nlm.nih.gov/) and then entered into the Swiss Target Prediction database (http://www.swisstargetprediction.ch/) and the PharmMapper database (http://www.lilab-ecust.cn/pharmmapper/) for target prediction corresponding to the compounds, with the species restricted to “*Homo sapiens*.” The filtering criterion for the Swiss Target Prediction database was as follows: The targets with a probability greater than 0 were saved (Tian et al., 2023; Zhi et al., 2023; Mohamed et al., 2023).

#### 2.1.2 Potential targets intersection of HQQD with IVP

Relevant genes associated with IVP were identified from multiple databases, including Genecards (https://www.genecards.org), OMIM (https://www.omim.org), DisGeNet (https://www.disgenet.org), and TTD (https://db.idrblab.org), using the MeSH keywords “influenza viral pneumonia” and “primary influenza virus pneumonia.”

The UniProt database (https://www.uniprot.org/) was employed to retrieve the official symbols for each protein target related to the identified chemicals and genes, with the species restricted to “*Homo sapiens*.” (Apweiler et al., 2004) Subsequently, an online bioinformatics platform (http://www.bioinformatics.com.cn) was utilized to construct the intersecting target network between HQQD and IVP (Bardou et al., 2014).

#### 2.1.3 Key target screening and network construction

To thoroughly investigate the molecular mechanism targets of HQQD in IVP treatment, the CytoHubba and CytoNCA plug-ins in Cytoscape software (ver. 3.9.0) were used to generate the protein-protein interaction (PPI) network. Topological analysis was conducted with six parameters, including network centrality, local average connectivity-based method, eigenvector centrality, closeness centrality, degree centrality, and betweenness centrality (BC). The filtering criterion for the core targets was as follows: The degree values were greater than the median of each parameter (Wang et al., 2023). The STRING database (https://string-db.org) was accessed to input the intersection targets, with “*Homo sapiens*” as the species, and the minimum interaction threshold was set to “highest confidence 0.900” to derive the final interaction network (Szklarczyk et al., 2019). Kyoto Encyclopedia of Genes and Genomes (KEGG) pathway analysis was conducted using the DAVID database (https://david.ncifcrf.gov/). (Wan et al., 2024) The resulting diagram from KEGG pathway analysis was generated using Cytoscape software (ver. 3.9.0) and the bioinformatics online platform (https://www.bioinformatics.com.cn/) (Tang et al., 2023).

### 2.2 Influenza a virus and experimental animals

The mouse-adapted IAV strain (H1N1/PR8) was propagated and maintained in the ABSL-2 biosafety laboratory at the Centre for Animal Experimentation, Guangzhou University of Traditional Chinese Medicine (Guangzhou, China). The virus was adapted for replication in BALB/c mouse lungs through eight sequential passages. The virus, replicated and plaque-purified in MDCK cells and subsequently replicated in 9-day-old chick embryos, was determined to have a median lethal dose (LD50) of 2 × 10^−15^/50 μL. The virus was then stored at −80°C until further use.

Male BALB/c mice (age: 36–42 days, weight: 18–22 g) were obtained from Zhuhai BesTest Bio-Tech Co., Ltd. (specific pathogen-free degree; certificate number: SCXK (yue) 2020-0051). All experimental procedures were conducted in accordance with the Declaration of Helsinki.

### 2.3 Preparation of therapeutic drugs

The composition of HQQD is detailed in Table 1. All Chinese medicine herbs were purchased from the First Affiliated Hospital of Guangzhou University of Chinese Medicine. Firstly, all crude drugs were soaked in 1.5 L water for 40 min, then they were decocted to boiling at 100°C for 40 min. The drugs were boiled once again for 30 min with 0.75 L water. The decoction was merged and filtered through four-layer gauze. The corresponding solution concentrations were 0.46 g/mL, 0.92 g/mL, and 1.85 g/mL. The decoction was then stored at 4°C for future use. The chemical constituents of the HQQD preparation were identified using UHPLC-Q/Orbitrap-MS chromatography, with the procedure and characteristic chromatogram presented in Supplementary Figure S1.

**TABLE 1:** The main drugs contained in the Haoqin Qingdan formula.

Plant names	Family	Genus	Authorities	Weight (g)
*Artemisia Annua L*	*Compositae*	*Artemisia*	Sweet Wormwood Herb	10
*Scutellariae Radix*	*Labiatae*	*Scutellaria*	Baical Skullcap Root	15
*Citri Reticulatae Pericarpium*	*Rutaceae*	*Citrus*	Dried Tangerine Peel	6
*Pinelliae Rhizoma*	*Araceae*	*Pinellia*	Pinellia Tuber	10
*Bambusae Caulis in Taenias*	*Poaceae*	*Bambusa*	Bamboo Shavings	10
*Poria*	*Polyporaceae*	*Wolfiporia*	Indian buead	15
*Aurantii Fructus*	*Rutaceae*	*Citrus*	Seville orange fruit	10
*Isatidis Folium*	*Brassicaceae*	*Isatis*	Indigowoad Leaf	15
*Isatidis Radix*	*Brassicaceae*	*Isatis*	Indigowoad Root	15
*Radix Glycyrrhizae*	*Fabaceae*	*Glycyrrhiza*	Liquorice Root	6
*Talcum*	*—*	*—*	Talcum	30

### 2.4 Establishment of an H1N1 infection model and treatment

Fifty male BALB/c mice were randomly assigned into five groups, with ten mice per group (Disease and Prevalence, 2016): control group (Cavallazzi and Ramirez, 2018), virus-infected group (VC) (Ryabkova et al., 2021), low-dose group (LD) (Lobo et al., 2019), medium-dose group (MD), and (Kalil and Thomas, 2019) high-dose group (HD). After a 3-day acclimatization period, virus inoculation commenced. On day 0, mice in groups (Cavallazzi and Ramirez, 2018)-(5) received intranasal injections of H1N1 virus at a dose of 2 LD50 to establish the virus-infected model. Subsequently, from days 0–4, model mice were administered oral medication once daily. Mice were maintained under a 12-h light/dark cycle at a temperature of 23°C ± 1°C and relative humidity of 50% ± 5%, with unrestricted access to food and water. Blood, lung, and colon tissues were collected on day 5 following anesthesia and sacrifice. The right upper lung lobe and a 1 cm segment of the colon were fixed in 4% paraformaldehyde for pathological evaluation while the remaining tissues were cryopreserved for further analysis.

### 2.5 Histopathological examination

Lung and colon tissues were fixed in 4% paraformaldehyde, dehydrated, and embedded in paraffin wax. Hematoxylin and eosin (H&E) staining was performed on 3 µm thick sections of paraffin-embedded tissues, which were then scanned using a panoramic scanning electron microscope to assess the images. Lung injury severity was graded on a scale of 0–4 based on four criteria: alveolar congestion, hemorrhage, leukocyte infiltration or neutrophil aggregation in the airspaces or blood vessel walls, and alveolar wall thickness. The total score was the sum of these individual scores (Tanaka et al., 2008).

### 2.6 Western blot analysis

Fresh lung tissues were subjected to protein extraction, quantification, SDS-PAGE electrophoresis, membrane transfer, blocking, incubation with primary and secondary antibodies, and chemiluminescence. Semi-quantitative analysis of protein expression was conducted using ImageJ software for grayscale scanning of the target and internal reference proteins to determine relative protein expression levels.

### 2.7 Quantitative real-time polymerase chain reaction

Total RNA was extracted from frozen lung samples using TRIzol reagent. The expression levels of TLR7 and MyD88 were measured *via* quantitative real-time PCR (qRT-PCR) utilizing the HiScript^®^ II Q RT SuperMix for qPCR Kit (Vazyme, Nanjing, China) under the following conditions: initial denaturation at 95°C for 3 min, followed by 45 cycles of 95°C for 10 s and 60°C for 30 s, with melting curve analysis from 65°C to 95°C, increasing by 0.5°C every 5 s, and a final extension at 95°C for 30 s. A BioRad CFX Connect™ system (Bio-Rad Laboratory, CA, United States) was employed for the qRT-PCR analysis. The primer sequences used are detailed in Table 2. The expression levels of all target transcripts were normalized to the housekeeping gene β-actin within the same tissue.

**TABLE 2:** The primers used for TLR7, MyD88 and β-actin.

Gene	RNA oligos	
TLR7	Forward	5′-ATG​TGG​ACA​CGG​AAG​AGA​CAA-3′
	Reverse	5′-GGT​AAG​GGT​AAG​ATT​GGT​GGT​G-3′
MyD88	Forward	5′-TCA​TGT​TCT​CCA​TAC​CCT​TGG​T-3′
	Reverse	5′-AAA​CTG​CGA​GTG​GGG​TCA​G-3′
β-actin	Forward	5′-GGC​TGT​ATT​CCC​CTC​CAT​CG-3′
	Reverse	5′-CCA​GTT​GGT​AAC​AAT​GCC​ATG​T-3′

### 2.8 Cytokines detection

Plasma and lung samples were collected from mice to assess cytokine levels. The concentrations of inflammatory cytokines, including IFN-γ, TNF-α, IL-6, IL-1β, and LPS (JL10967, JL10484, JL20268, JL18442, JL20691; JiangLai Biotechnology, Shanghai, China), were quantified using commercially available ELISA kits according to the manufacturer’s instructions. Absorbance was measured at 450 nm using a Bio-Tek Synergy H1 microplate reader.

### 2.9 Immunohistochemistry assay of the relative protein expression

Paraffin-embedded lung specimens were sectioned into 3 µm thick slices using a microtome. After drying, dewaxing, dehydration, antigen retrieval, and quenching of endogenous peroxidase activity, the sections were blocked with 5% BSA for 30 min. The samples were then incubated overnight at 4°C with antibodies against NF-κB p65 and p-p65 (8242S, CST, United States; ab131100, Abcam, United Kingdom). This was followed by incubation at 37°C with an HRP-conjugated goat anti-rabbit IgG antibody. The slides were subsequently stained using the DAB chromogenic substrate solution. Images were captured using a panoramic scanning electron microscope, with five fields observed per sample. The average optical density (AOD), calculated as the integrated optical density (IOD) over the positive area, was determined.

### 2.10 16S rRNA gene sequencing

Using 16S rRNA sequencing, the diversity of cecal intestinal flora was analyzed in five randomly selected fecal samples from each group. Total bacterial DNA was extracted, and its concentration and purity were measured using a NanoDrop One spectrophotometer (Thermo Fisher Scientific, MA, United States). The V3-V4 region of the 16S rRNA gene was then amplified using specific primers for polymerase chain reaction (PCR). The forward primer “ACT​CCT​ACG​GGA​GGC​AGC​A” and the reverse primer “GGACTACHVGGGTWTCTAAT” were employed to amplify these regions. Primers with a 12 bp barcode were synthesized by Invitrogen (Carlsbad, CA, United States). The quality of the PCR products was evaluated using 1% agarose gel electrophoresis, and the PCR products were subsequently purified with an E. Z.N.A. Gel Extraction Kit (Omega, United States).

Following the manufacturer’s instructions, index codes were added, and sequencing libraries were constructed using the NEBNext^®^ UltraTM II DNA Library Prep Kit for Illumina^®^ (New England Biolabs, MA, United States). Library quality was assessed using a Qubit^@^ 2.0 Fluorometer (Thermo Fisher Scientific, MA, United States). The library was ultimately sequenced on an Illumina Nova6000 platform, and sequences were referenced using the Silva 132 database. Operational taxonomic unit (OTU) clustering analysis was conducted with Usearch v10.0.240 at a 97% sequence similarity threshold. Subsequent analyses were performed based on these results.

### 2.11 Statistical analysis

All statistical analyses were conducted using GraphPad Prism 9 software (Inc., CA, United States). Data fitting a normal distribution underwent a homogeneity of variance test, and if homogeneity was confirmed, one-way analysis of variance (ANOVA) was performed for comparisons among multiple groups. A *P*-value of <0.05 was considered statistically significant.

## 3 Results

### 3.1 HQQD therapy reduced the levels of intestinal and pulmonary damage in the model mice

**FIGURE 1:**
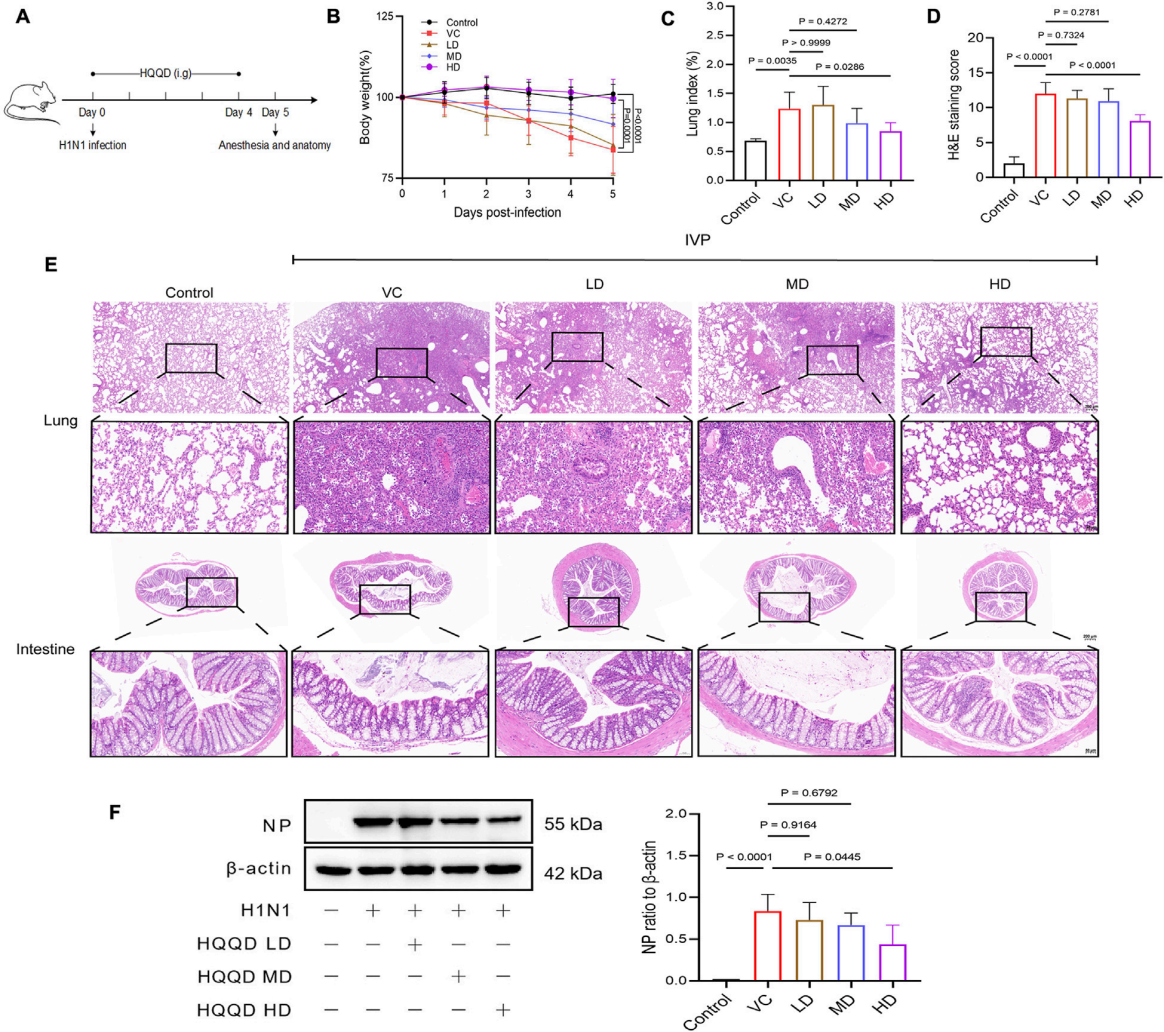
Evaluation of the efficacy of HQQD Decoction. **(A)** Schematic diagram of the drug efficacy experiment. **(B)** Body weight trend chart following virus infection. **(C)** Lung indices in the validation experiment. **(D)** HE staining scores of lung tissue in mice. **(E)** HE staining images of lung and intestine tissues in mice (scale bar: 200 and 50 μm). **(F)** Western blot analysis showing the inhibitory effect of HQQD against influenza virus (n = 4). Data are presented as means ± standard errors of the means.

Following IAV infection, the IVP model was successfully established (Figure 1A). Six days post-infection, the VC group exhibited a significant reduction in body weight compared to the control group, whereas the HD group maintained a notably higher body weight than the VC group (*P* < 0.01), indicating that HD treatment provided protective effects on weight in mice (Figure 1B).

The lung index was significantly elevated in the VC group compared to normal mice but was markedly reduced in the HD treatment group (*P* < 0.05; Figure 1C). Histopathological analysis using H&E staining revealed severe structural distortion in the lungs of the VC group, including hyperplasia of connective tissue and deformation of the bronchi, alveoli, and alveolar tubes. There was also notable infiltration of inflammatory cells in the pulmonary interstitium, an abundance of red blood cells in the lung vasculature, and exudation in the bronchial lumen. In contrast, the HD group showed significantly less lung damage compared to the VC group (*P* < 0.01). Additionally, pathogenic factors induced gut mucosal damage, evidenced by the infiltration of inflammatory cells, villus separation, significantly reduced villi, and a disrupted brush border in the VC group, which were not observed in the control group. The HD intervention mitigated these intestinal damages compared to the VC group (Figures 1D, E). Furthermore, NP protein expression in the lungs was significantly decreased in the HD group (*P* < 0.05; Figure 1F).

### 3.2 Potential therapeutic mechanisms by which HQQD acts on influenza

**FIGURE 2:**
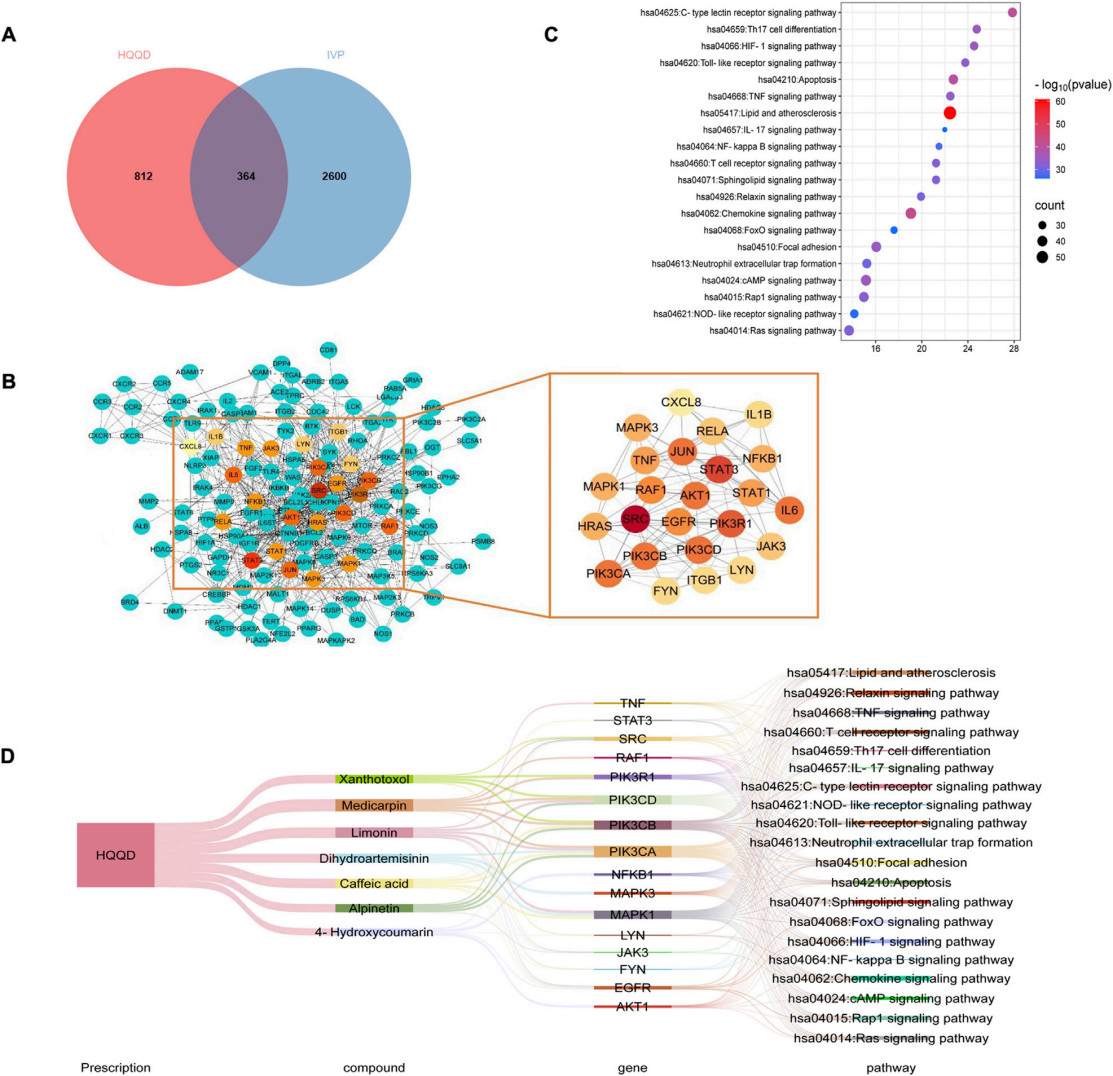
Results of Network Analysis. **(A)** Venn diagram illustrating 364 common targets between HQQD and IVP. **(B)** PPI network diagram depicting protein interactions, where targets are represented by nodes and protein-protein interactions by edges. The size and color of nodes are governed by degree centrality, with larger nodes and red underpainting indicating higher degree centrality. **(C)** Top 20 signaling pathways identified through KEGG enrichment analysis, with larger points indicating greater gene enrichment. **(D)** Sankey diagram of “formula-components-targets-pathways”. The lines represent the properties of targets and pathways.

UHPLC-MS analysis identified 236 chemical constituents in HQQD, which were linked to 1176 compound-related targets. Supplementary Table S1 provided detailed information on these candidate bioactive ingredients. Additionally, 2964 IVP-associated genes were extracted from the Genecards, OMIM, DisGeNET, and TTD databases. The overlapping targets were visualised using a Venn diagram. Of these intersecting targets, 364 targets were closely associated with the treatment of IVP (Figure 2A). To further elucidate the PPI of HQQD, datasets were processed using Cytoscape 3.9.0 software to construct a PPI network. Moreover, the MCC method was employed to identify key hub target genes, enhancing screening accuracy (Chin et al., 2014). The primary genes ranked by BC were considered as hub targets, such as STAT3, TNF, IL-6, and IL-1β (Figure 2B). Furthermore, the top 20 pathways obtained by KEGG enrichment analysis were showed in Figure 2C (*P* < 0.05) (Huo et al., 2022). Then, the formula-components-targets-pathways network between HQQD and IVP contained 7 core components, 16 potential targets, and 20 related pathways (Figure 2D). (Wan et al., 2024; Wu et al., 2024) The toll-like receptor signaling pathway and the TNF signaling pathway were particularly associated with HQQD’s mechanism in treating IVP. There are 7 notable compounds correspond to core targets and potential mechanisms for pathways: caffeic acid, limonin, medicarpin, alpinetin, xanthotoxol, 4-hydroxycoumarin, and dihydroartemisinin, which may be closely related to the Protective effect of HQQD.

### 3.3 HQQD regulated the TLR7/MyD88/NF-κB signaling pathway in lung tissue

Host cells initiate an immune response against the influenza virus by activating both innate and adaptive immunity upon recognition of the virus. Toll-like receptors (TLRs), a class of pattern recognition receptors primarily expressed in antigen-presenting cells, play a pivotal role in both acquired and innate immunity. TLR7, a receptor for single-stranded RNA (ssRNA), is located in the membranes of endosomes, particularly in phagocytic cells like macrophages. It is essential for recognizing the influenza virus and triggering the secretion of inflammatory cytokines from neutrophils (Diebold et al., 2004). Upon binding to ssRNA, TLR7 activates a unique MyD88-dependent pathway, leading to the activation of NF-κB family members, which subsequently promote the production of IFN-α/β (Akira et al., 2006). NF-κB mediates the host antiviral immune response by translocating to the nucleus, where it stimulates the synthesis of various cytokines (Jiang and Zhang, 2021).

**FIGURE 3:**
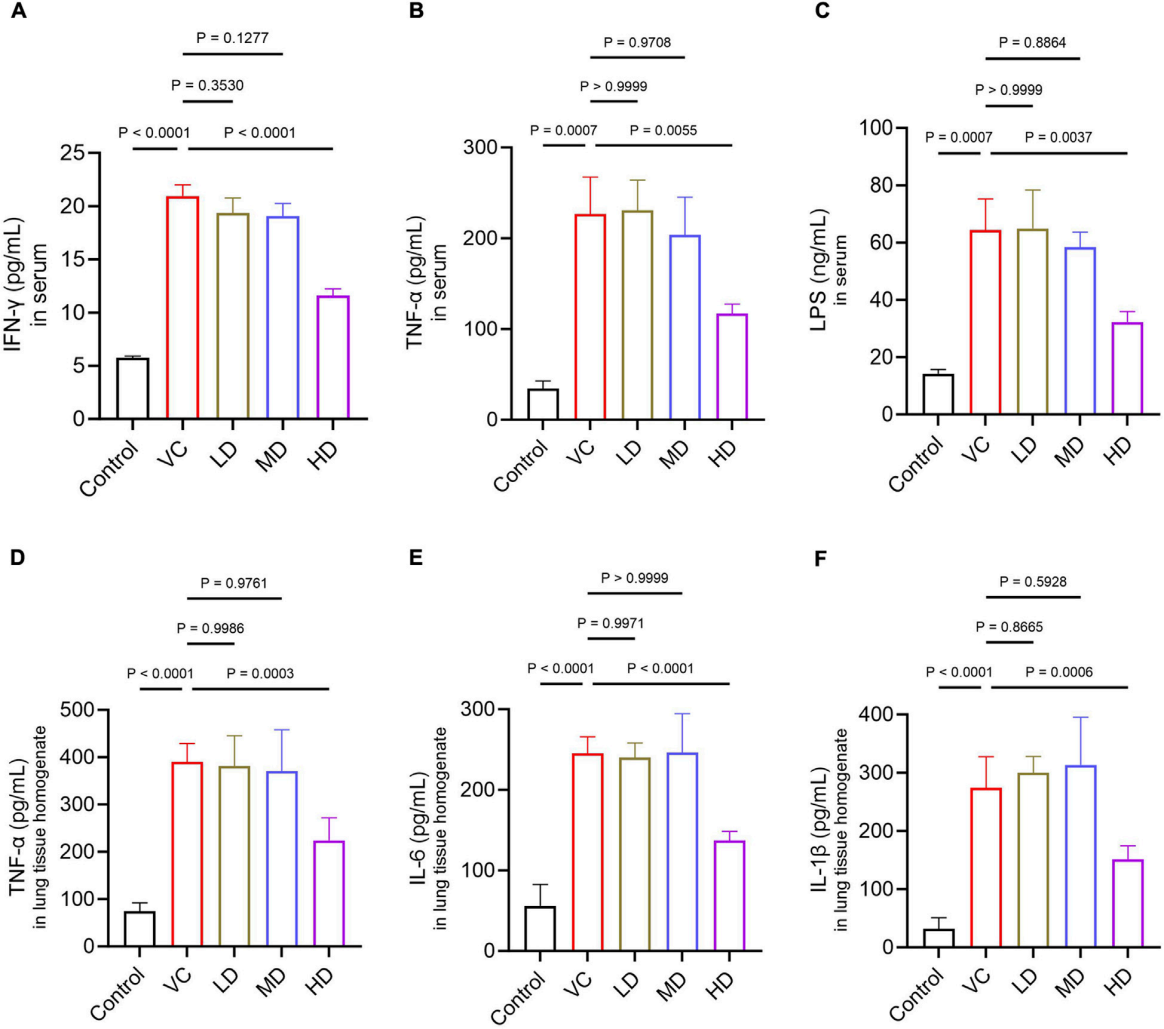
Effect of the HQQD on serum and lung cytokine levels. **(A)**, **(B)**, **(C)** ELISA analysis of serum inflammatory factor levels (IFN-γ, TNF-α, and LPS) in mice (n = 6). **(D)**, **(E)**, **(F)** ELISA analysis of lung inflammatory factor levels (TNF-α, IL-6, and IL-1β) in mice (n = 6). Data are presented as means ± standard errors of the means.

To assess cytokine levels, plasma and lung tissues were collected from the mice. When the permeability of intestinal wall cells is increased and their structure compromised, LPS, a product of gut bacteria, can translocate from the gut into the circulatory system (Liu et al., 2020). The serum of the VC group exhibited a substantial increase in the release of IFN-γ, TNF-α, and LPS (*P* < 0.01; Figures 3A–C). The release levels of TNF-α, IL-6, and IL-1β in the lung of the VC group were significantly increased (*P* < 0.01; Figures 3D–F). In contrast, HD treatment significantly inhibited the secretion of these inflammatory factors compared to the VC group (*P* < 0.01). Overall, the findings indicated that high-dose HQQD treatment effectively suppressed the inflammatory response in the lungs and colons of IVP mice.

**FIGURE 4:**
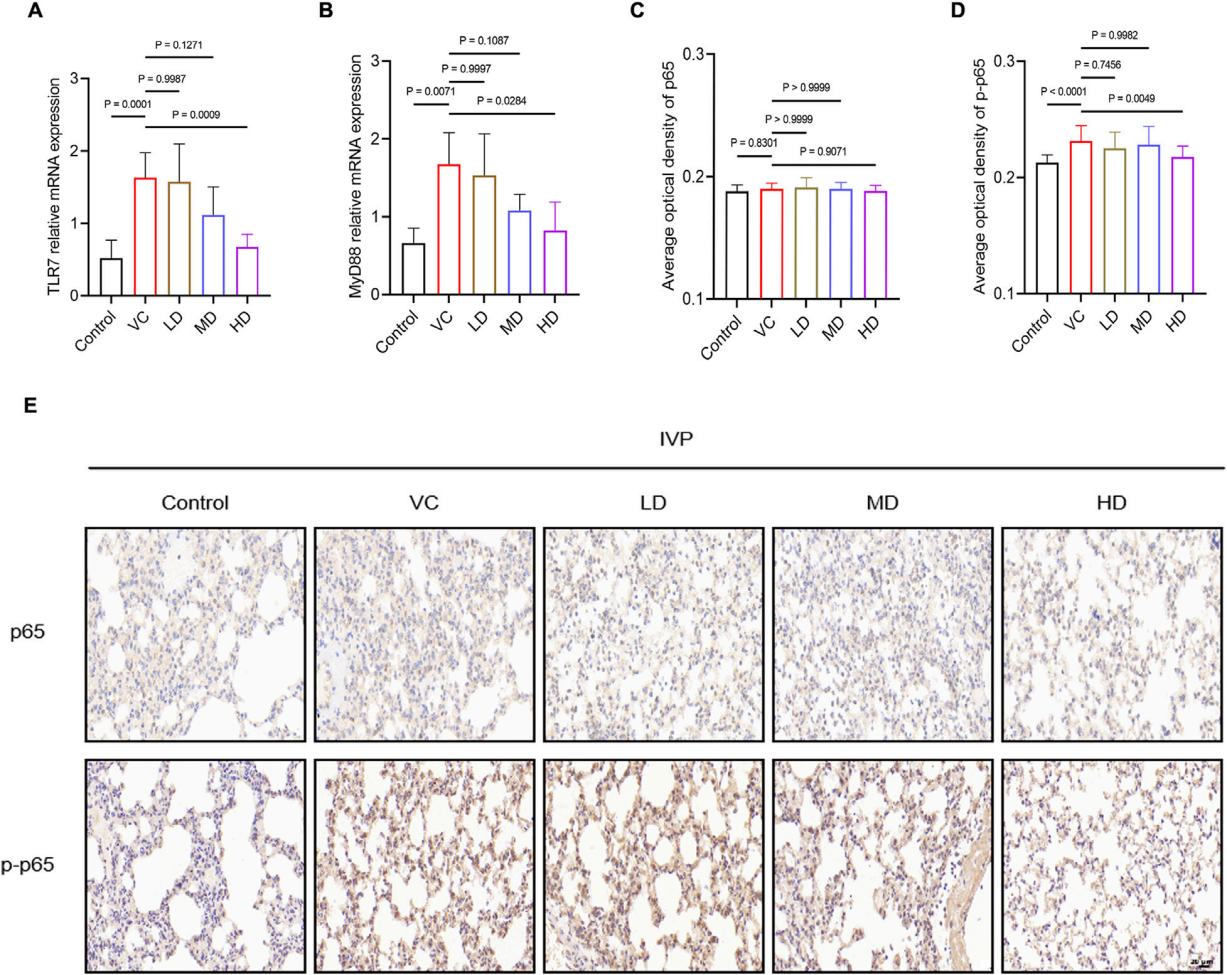
Treatment Mechanism of HQQD for IVP. **(A)**, **(B)** Expression levels and quantitative analysis of TLR7 and MyD88 mRNA in lung homogenates (n = 6). **(C)**, **(D)**, **(E)** Immunohistochemical staining and AOD calculation of NF-κB p65 and p-p65 in lung tissue of model mice (scale bar: 50 μm; n = 4). Data are presented as means ± standard errors of the means.

The results also showed that mRNA expression levels of TLR7 and MyD88 were significantly elevated in the lung tissues of the VC group but were markedly downregulated in the HD group (*P* < 0.05; Figures 4A, B). Additionally, there was a significant increase in p-p65 protein expression in the VC group, which was reduced in the HD-treated group (*P* < 0.01; Figures 4C–E). In conclusion, high-dose HQQD may effectively mitigate the overactive immune response by downregulating the expression of key mRNAs and proteins in the TLR7/MyD88/NF-κB p65 signaling pathway.

### 3.4 HQQD improved the abundance and structure of the gut microbiota

**FIGURE 5:**
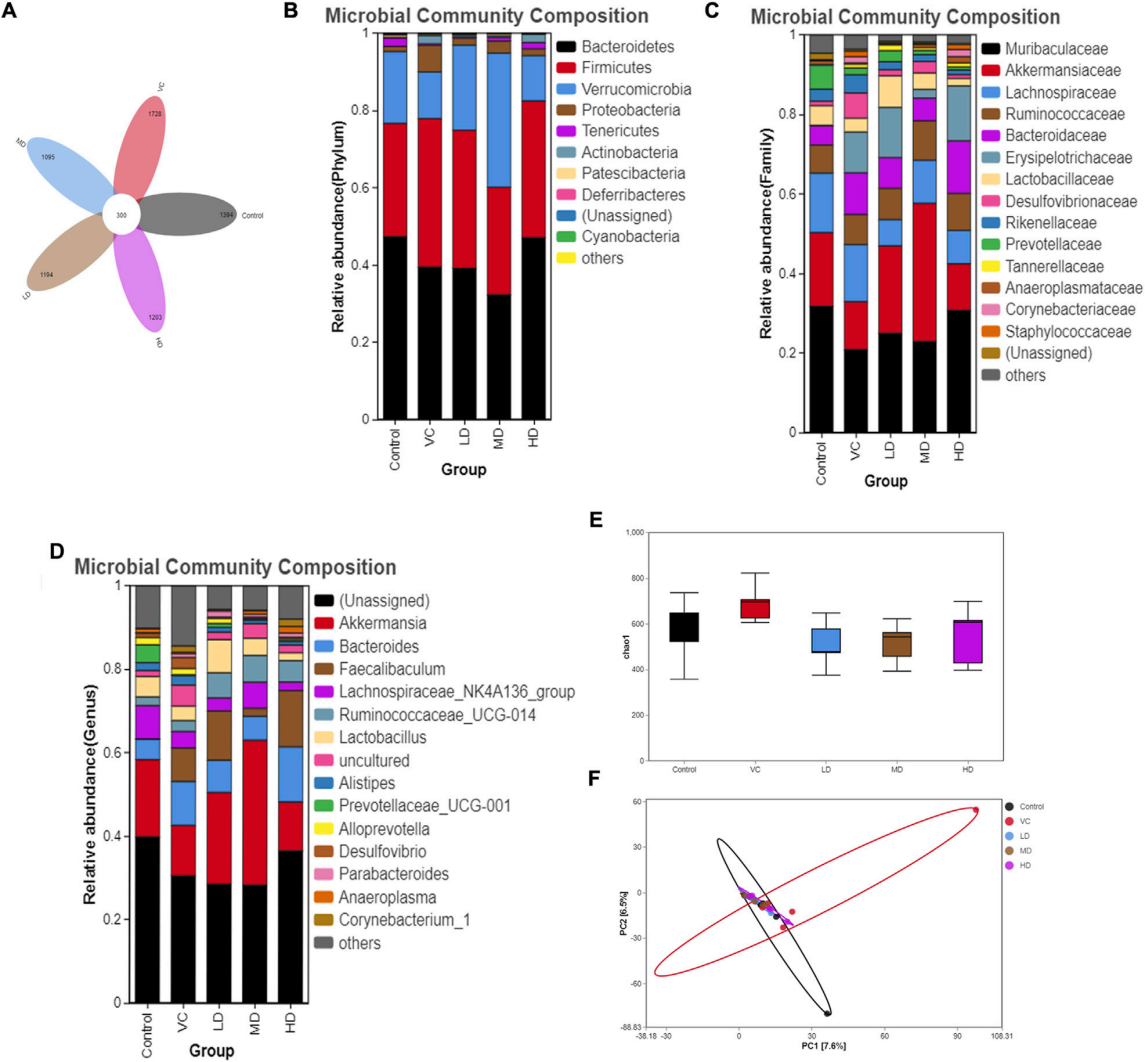
Intestinal Flora Diversity Analysis on Day Six. **(A)** Venn diagram showing different strains identified through OTU analysis. **(B)** Relative abundance and composition of microbial communities at the phylum level. **(C)** Relative abundance and composition of microbial communities at the family level. **(D)** Relative abundance and composition of microbial communities at the genus level. **(E)** Alpha diversity analysis represented by the Chao1 index. **(F)** PCA analysis showing differences between samples at the OTU level.

OTU data were obtained by sequencing five samples per group, with sequences classified as OTUs based on a similarity threshold of >97% through bioinformatics analysis. The control group exhibited 1,394 OTUs, the VC group 1,728 OTUs, the LD group 1,194 OTUs, the MD group 1,095 OTUs, and the HD group 1,203 OTUs. A total of 300 OTUs were shared across all five groups (Figure 5A).

At the phylum level, and based on a criterion of total relative abundance exceeding 95%, the dominant microbiomes were identified as *Bacteroidetes*, *Firmicutes*, *Verrucomicrobia*, and *Proteobacteria*. Figures 5B–D reveal that *Proteobacteria* proportions significantly increased following H1N1 infection (*P* < 0.05). However, high-dose treatment led to a significant downregulation in the proportions of *Proteobacteria* and *Deferribacteres* (*P* < 0.05). No significant variations in gut flora were observed at the family or genus levels among the five groups.

Alpha diversity analysis based on OTUs showed no significant differences between the VC and HD groups (*P* > 0.05; Figure 5E). Principal component analysis (PCA) indicated that while the gut microbiota of the HQQD-treated group resembled that of the control group, the VC group’s microbiota significantly diverged from the control group (Figure 5F).

**FIGURE 6:**
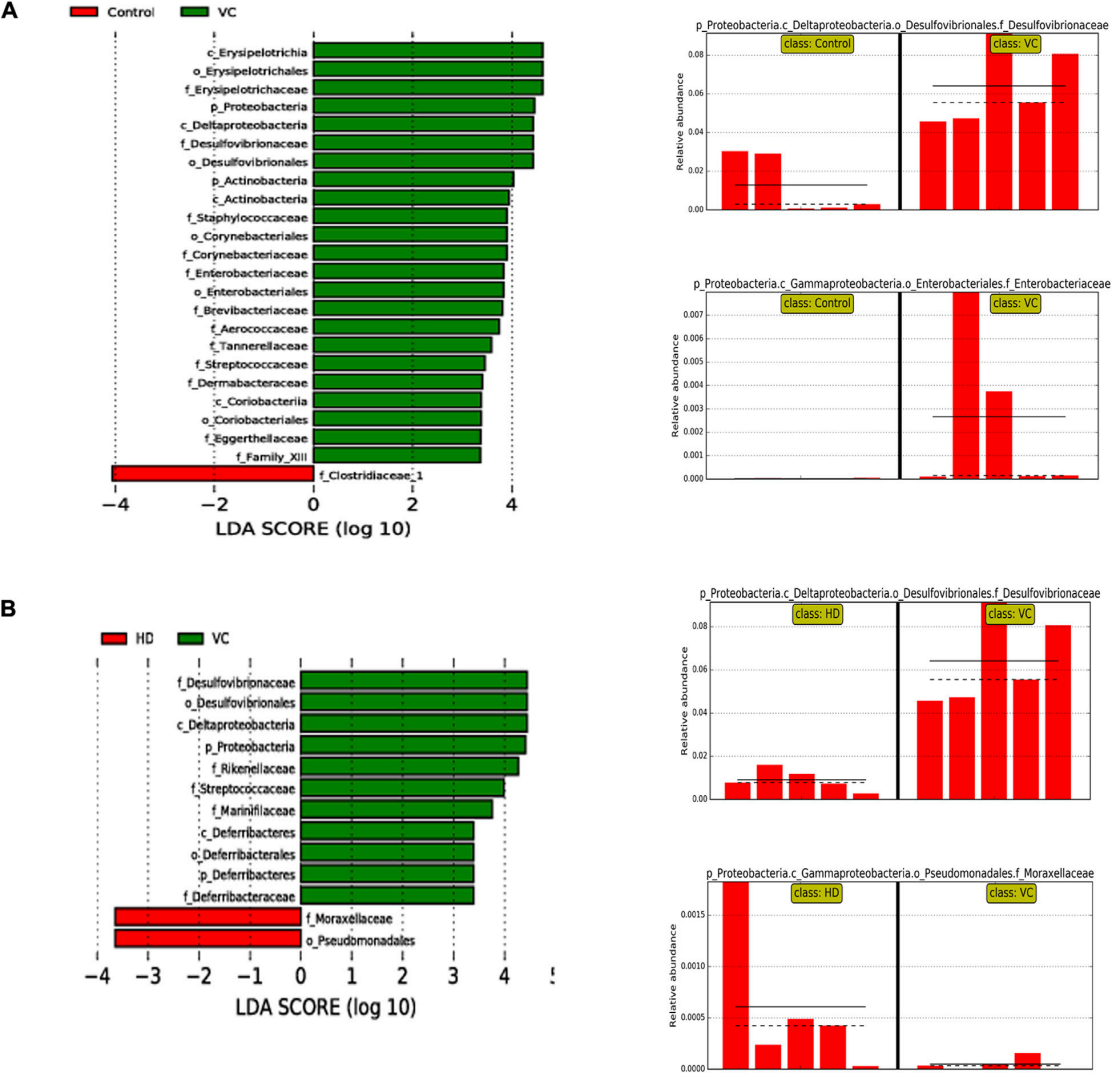
LEfSe Analysis of Intestinal Flora on Day Six. **(A)** LEfSe analysis at the phylum and family levels in the VC and control groups. **(B)** LEfSe analysis at the phylum and family levels in the VC and HD groups.

Community structure variations were further assessed using linear discriminant analysis effect size (LEfSe). The bar graph highlighted species with statistically significant differences in abundance between the groups (*p* < 0.05). The results demonstrated that, compared to the control group, the VC group exhibited significant over-representation of *Erysipelotrichaceae*, *Staphylococcaceae*, *Aerococcaceae*, *Streptococcaceae*, *Family_XIII*, *Desulfovibrionaceae*, *Enterobacteriaceae*, *Corynebacteriaceae*, *Brevibacteriaceae, Eggerthellaceae*, *Dermabacteraceae*, *Tannerellaceae*, *Proteobacteria*, and *Actinobacteria*. These changes were associated with damage to the intestinal wall barrier and a notable increase in pathogenic bacteria (*P* < 0.05; Figure 6A). In contrast, the HD group showed upregulation of *Moraxellaceae* and downregulation of *Deferribacteraceae*, *Desulfovibrionaceae*, *Rikenellaceae*, *Marinifilaceae*, and *Streptococcaceae* (*P* < 0.05; Figure 6B). Overall, H1N1 infection resulted in an upregulation of *Proteobacteria*, primarily within *Enterobacteriaceae* and *Desulfovibrionaceae*, with the HD group significantly downregulating *Desulfovibrionaceae* compared to the VC group.

## 4 Discussion

The current research elucidates the underlying mechanisms by which HQQD treats influenza virus-infected mice. HQQD, a traditional Chinese herbal preparation, has shown potential in treating pulmonary illnesses, including COVID-19 (Zhang et al., 2013). Previous studies have suggested its efficacy in this regard (Luo et al., 2016; Zhang et al., 2013). Given the complexity of TCM and the intricate composition of prescriptions, the application of network analysis and 16S rRNA bacterial gene sequencing has proven to be effective in uncovering the specific treatment mechanisms of traditional Chinese remedies. By employing these omics technologies, this study systematically elucidated the fundamental mechanisms through which HQQD addresses lung and gut damage caused by IVP, with findings validated through animal experiments. The results indicate that HQQD’s therapeutic effects are dose-dependent, significantly enhancing body weight, reducing lung indices and NP protein expression, mitigating inflammatory cytokine storms mediated by the TLR7/MyD88/NF–κB p65 signaling pathway, and restoring disordered flora.

The construction of PPI networks identified 24 intersecting targets relevant to IVP therapy. The combined data highlighted TNF, IL-6, and IL-1β as important targets in regulating immunity and inflammation. Through a formula-components-targets-pathways network analysis, we identified 7 potential core components. Several components have been shown to have antiviral and anti-inflammatory properties. Caffeic acid had direct antiviral effects and inhibited the multiplication of influenza A virus *in vitro* (Utsunomiya et al., 2014). Limonin, alpinetin, and 4-hydroxycoumarin had the potential to reduce LPS-induced acute lung injury by suppressing inflammation (Liang et al., 2023; Zhang and Ma, 2024; Li et al., 2024). Dihydroartemisinin has been shown to mitigate theinflammatory cytokine storm *via* the suppression of the NF-κB signaling pathway (Huang et al., 2019). In future work, we will further screen other potential active ingredients in the HQQD decoction and clarify whether these components have direct antiviral or anti-inflammatory effects. Furthermore, KEGG pathway analysis identified the toll-like receptor signaling pathway and TNF signaling pathway as primary targets of HQQD in IVP treatment. The combined analysis of the KEGG pathway and PPI data suggests that HQQD’s mechanisms are closely linked to the regulation of immunological, antiviral, and anti-inflammatory processes.

TLR7 recognizes the ssRNA genome of influenza viruses, triggering cytokine release, inflammatory responses, and NF-κB p65 activation through MyD88 (Arora et al., 2019). MyD88 is a key adaptor molecule in the TLR signaling pathways and a key node for downstream signal transduction (Jiang and Zhang, 2021), leading to the activation of NF-κB, which initiates the inflammatory cascade. In its resting state, NF-κB is inactive, but upon stimulation, it becomes active, producing p-NF-κB, which translocates from the cytosol to the nucleus to regulate transcription and promote the release of various inflammatory factors (Mulero et al., 2019). Validation experiments in IVP mice demonstrated that HQQD significantly reduced the release of inflammatory cytokines in plasma and lung, such as IL-6, IL-1β, TNF-α and IFN-γ. Moreover, high-dose HQQD therapy downregulated the mRNA expression levels of TLR7 and MyD88, as well as the protein expression level of p-NF-κB p65 in lung tissues.

The complex microbial ecosystem known as gut flora is essential for maintaining host health (Lane et al., 2023). The physiological effects of gut flora on the host are mediated by microbial metabolites (Li et al., 2014). Increasing evidence suggests that gut flora plays a protective role in the lungs against viral infections by modulating the innate immune response (Dumas et al., 2018). Our findings indicated that IVP disrupted microbiota balance and increased gut barrier permeability. Following H1N1 infection, the proportion of *Proteobacteria* increased, particularly within *Enterobacteriaceae* and *Desulfovibrionaceae*. In contrast, the HD group showed significant downregulation of *Desulfovibrionaceae* and *Moraxellaceae* compared to the VC group. Alterations in intestinal flora can directly impair intestinal tight junction proteins and indirectly compromise intestinal integrity by disrupting phosphorylation and dephosphorylation processes, leading to elevated levels of LPS in systemic circulation (Stephens and von der Weid, 2020). LPS from *Enterobacteriaceae* has been shown to exacerbate intestinal damage and increase permeability (Zeng et al., 2017). *Desulfovibrio*, the predominant genus within *Desulfovibrionaceae*, is a major sulfate-reducing bacterium (SRB) in the gut (Christophersen et al., 2011). SRBs consume short-chain fatty acids, crucial for intestinal epithelial cells, and produce hydrogen sulfide, which can damage intestinal epithelial cells. Additionally, LPS produced by these bacteria strongly induces inflammation (Lu et al., 2023; Wei et al., 2015).

It is well-established that LPS produced by intestinal bacteria can aggravate intestinal damage, increase permeability, mediate elevated LPS levels in the intestinal lumen and systemic circulation, and trigger systemic proinflammatory and immunomodulatory responses (Di Lorenzo et al., 2019; Mao et al., 2020). Dysbiosis of intestinal flora and compromise of the intestinal epithelial barrier can allow LPS from pathogenic bacteria to enter systemic circulation, activating innate immunity in the lungs and exacerbating inflammatory injury. Once TLRs in alveolar capillary endothelial and epithelial cells are activated, the downstream TLR/NF-κB signaling pathway is triggered, leading to the upregulation of genes associated with inflammation. Studies have shown that intestinal dysbiosis in influenza virus-infected mice causes severe damage to lung and intestinal tissues, whereas restoring intestinal microbiota can alleviate inflammation and pneumonia *via* the TLR7 signaling pathway (Gao et al., 2023). Previous research underscores the importance of intestinal flora recovery and innate immune responses in pathogen defense and maintaining immune system homeostasis (Fu et al., 2018). In summary, disruption of intestinal microbiota weakens the mucosal barrier, allowing LPS to enter systemic circulation and activate the lung TLR/NF-κB signaling pathway, potentially worsening lung inflammation. Our research suggests that high-dose HQQD treatment may regulate gut microbiota and preserve the intestinal epithelial barrier’s function. By reducing the overabundance of pathogenic bacteria and limiting endotoxin entry into systemic circulation, HQQD likely exerts its therapeutic effects on pneumonia by modulating the gut-lung axis, closely linked to lowering intestinal barrier permeability and eliminating harmful microorganisms.

## 5 Conclusion

In summary, our findings provide a theoretical foundation for the protective effects of HQQD against IVP. By inhibiting NP protein expression and blocking the activation of the TLR7/NF-κB signaling pathway, HQQD may reduce the production of inflammatory mediators. Additionally, HQQD combats IVP by enhancing gut flora diversity and preserving the function of the intestinal epithelial barrier. This process also lowers the levels of LPS entering systemic circulation and suppresses excess pathogenic microbes. Our research confirms that the antiviral, anti-inflammatory, and gut homeostasis-regulating properties of HQQD are crucial to its efficacy in treating respiratory disorders, offering experimental support for the Chinese medicine theory of “simultaneous pulmonary and intestinal therapy.” Building on these results, further exploration into additional treatment mechanisms will be conducted using a mouse model of antibiotic-induced gut microbiota dysbiosis combined with the active components of HQQD.

## Data Availability

The original contributions presented in the study are publicly available. The data have been deposited in the Sequence Read Archive (SRA), accession number SRP548198. These SRA data can be found here: https://www.ncbi.nlm.nih.gov/sra/?term=SRP548198.

## Abbreviations

HQQD, Haoqin Qingdan decoction; IL-6, interleukin-6; IL-1β, interleukin-1β; IFN-γ, interferon-gamma; TNF-α, tumor necrosis factor-alpha; IVP, influenza viral pneumonia; UHPLC-MS, ultra-high-performance liquid chromatography coupled with mass spectrometry; IAV, influenza A virus; TCM, traditional Chinese medicine; COVID-19, Coronavirus Disease 2019; PPI network, protein-protein interaction network; BC, betweenness centrality; KEGG, Kyoto Encyclopedia of Genes and Genomes; LD50, median lethal dose; VC, virus-infected group; LD, low-dose group; MD, medium-dose group; HD, high-dose group; H&E, hematoxylin and eosin; qRT-PCR, quantitative real-time polymerase chain reaction; LPS, lipopolysaccharide; p-p65, phospho-NF-κB p65; AOD, average optical density; OTU, operational taxonomic unit; TLRs, Toll-like receptors; ssRNA, single-stranded RNA; PCA, principal component analysis; LEfSe, linear discriminant analysis effect size.
