# Supplementary figures and images for: Protective effect of Haoqin Qingdan decoction on pulmonary and intestinal injury in mice with influenza viral pneumonia
Source: Front Pharmacol. 2024 Dec 6;15:1449322. doi: 10.3389/fphar.2024.1449322 (PMC11658977; doi:10.3389/fphar.2024.1449322)

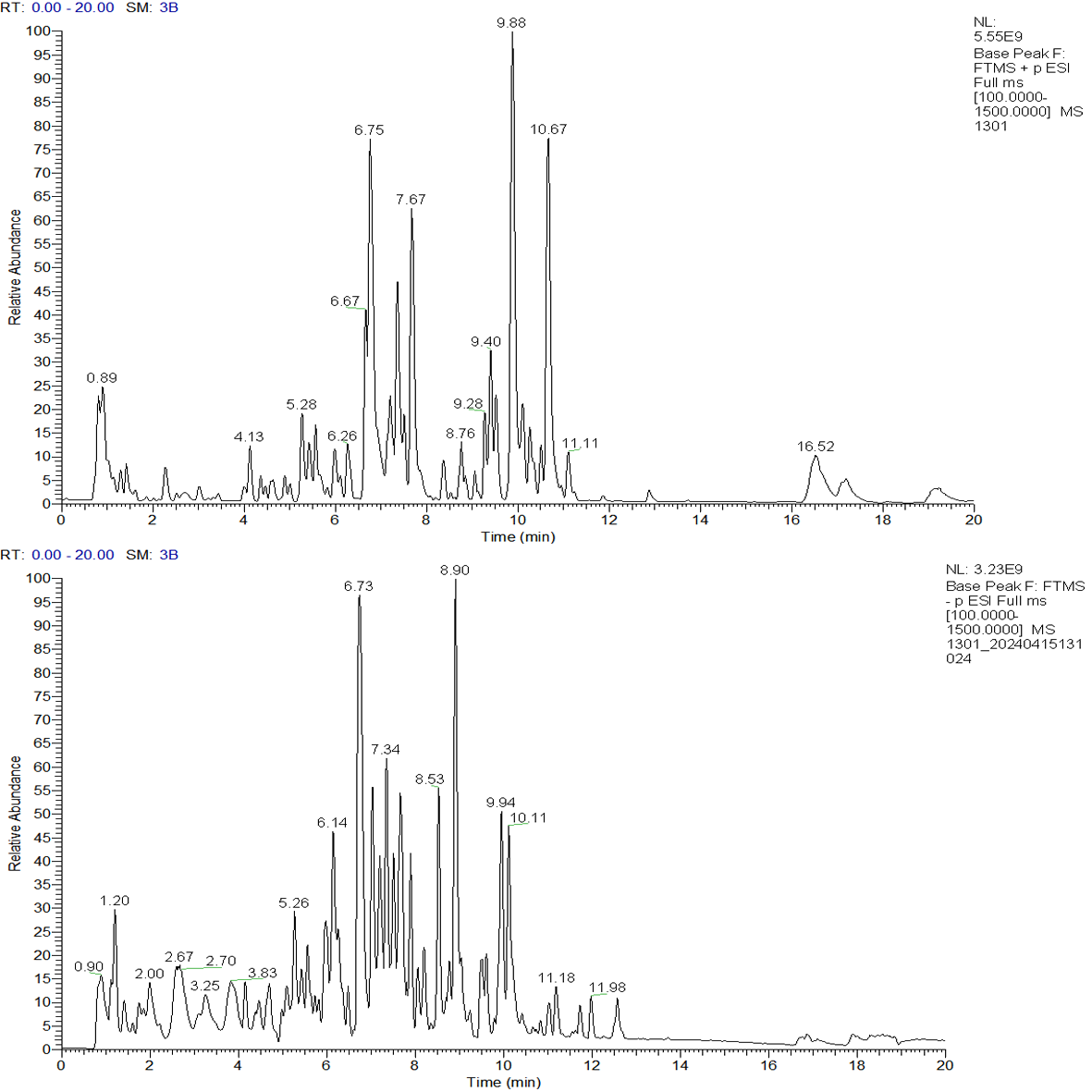

Supplement: Supplementary file 1 [file Image1.TIF]
