# Supplementary material for: Protective effect of Haoqin Qingdan decoction on pulmonary and intestinal injury in mice with influenza viral pneumonia
Source: Front Pharmacol. 2024 Dec 6;15:1449322. doi: 10.3389/fphar.2024.1449322 (PMC11658977; doi:10.3389/fphar.2024.1449322)
Supplement: Supplementary file 2 [file DataSheet1.docx]

Supplementary Material

**1 Supplementary Figure**

**
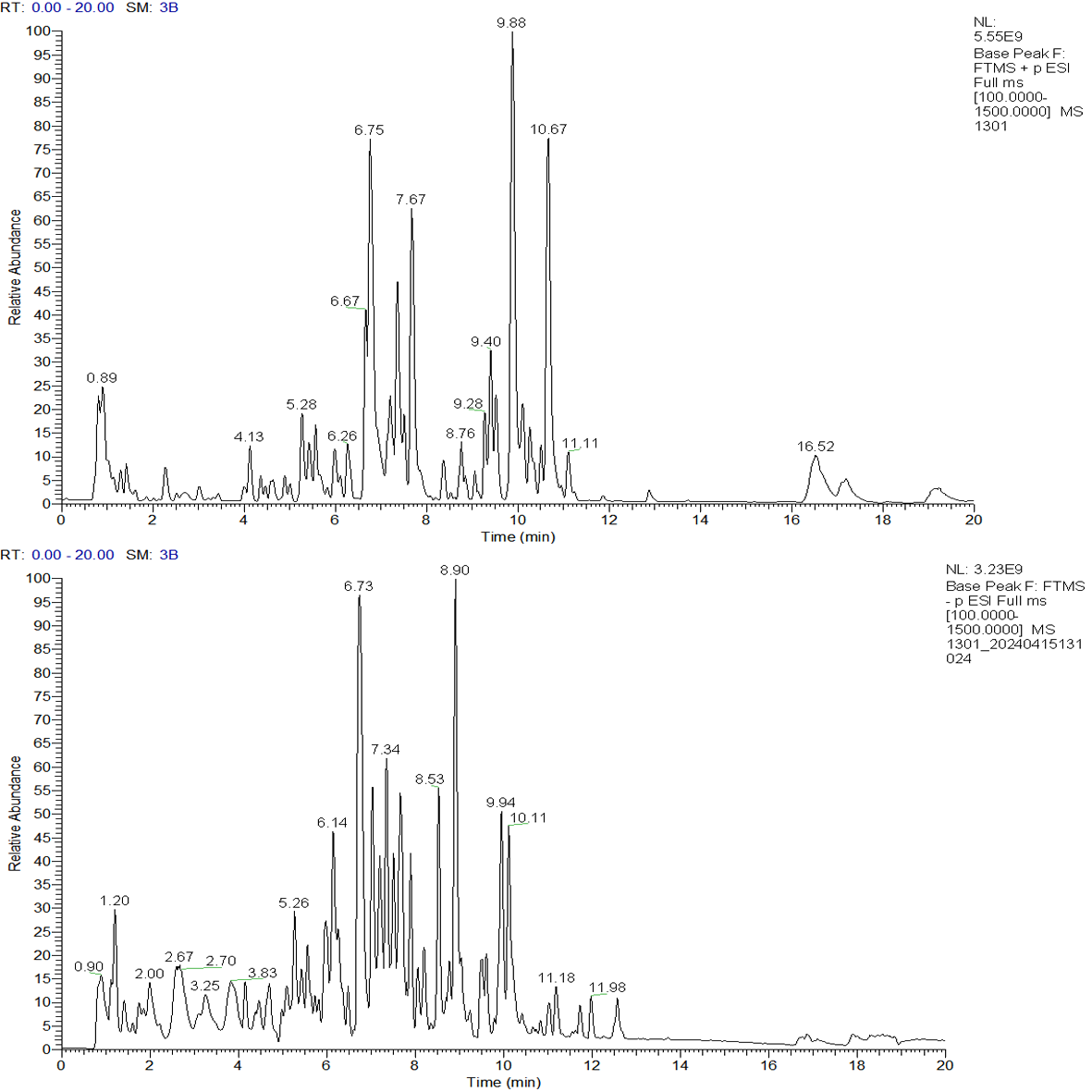
**

**Supplementary Figure 1| Total ion current diagram of HQQD.** (A) The above figure means total ion flow diagram of the positive ion pattern. (B) The below figure means total ion flow diagram of the negative ion mode.

The UHPLC-MS analysis procedure is as follows: 200 µL solution was added into 200 µL methanol, vortex for 10 min. After centrifuged for 10 min at 13000 rpm and 4℃, The supernatant was subjected to the computer analysis. The liquid chromatograph is UltiMate 3000 UHPLC and the column is Thermo Hybrid C18 (1.9 μm, 2.1 mm×100 mm). Analysis was performed using a flow rate of 0.3 ml/min and an injection volume of 10 μL. The mobile phase is 0.1% formic acid / acetonitrile (B) -0.1% formic acid / water (A). The gradient elution procedure of the solution as follows: [t: 0 - 10 min, A: 90%, B: 10%; t: 10 - 15 min, A: 0%, B: 100%; t: 15 - 17.1 min, A: 90%, B: 10%; t: 17.1 - 20 min, A: 90%, B: 10%]. The mass spectrometry conditions are as follows: Q-Exactive HESI source (Thermo Fisher Scientific, CA, USA); Ion source temperature: 310℃; Capillary temperature: 320℃; Sheath gas flow rate: 30 units; Auxiliary air flow rate: 10 units; Positive ion mode spray voltage: 3 kV; Negative ion mode spray voltage: 2.8 kV. Data-dependent scanning analysis (DDA) was performed with a loop count set value of 10 and HCD energy using step-wise normalized collision energy with a set value of 10, 28, 35 eV. The primary MS scan range was 100 - 1,500 m/z with resolution set to 70,000, AGC target set to 3E6, and injection time set to 200 ms. The secondary MS resolution was set to 17,500, AGC target was set to 1E5 and injection time to 50 ms. The collected data were initially searched by Compound Discoverer (V 3.2, Thermo Fisher Scientific, CA, USA). It included the chemspider plant database CHEBI, CHEMBL, natural product database, OTC Traditional Chinese medicine database and mz cloud database to analyze the possible chemical composition and retain the chemical composition.

**2 Supplementary Table**

**Supplementary Table 1| Chemical constituents of HQQD identified by UHPLC-MS analysis**

| **No.** | **Detection mode** | **Ingredient** | **Molecular formula** | **Molecular weight** | **RT (min)** |
| --- | --- | --- | --- | --- | --- |
| 1 | P | Baicalin | C21H18O11 | 446.0835 | 6.76 |
| 2 | P | Tangeretin | C20H20O7 | 372.1202 | 10.67 |
| 3 | P | Wogonoside | C22H20O11 | 460.0995 | 7.68 |
| 4 | P | Isomeranzin | C15H16O4 | 260.1042 | 9.4 |
| 5 | N | oroxindin | C22H20O11 | 460.101 | 7.68 |
| 6 | N | Wogonin | C16H12O5 | 284.0688 | 9.94 |
| 7 | N | biochanin A | C16H12O5 | 284.0686 | 7.71 |
| 8 | P | Stachydrine | C7H13NO2 | 143.0944 | 0.89 |
| 9 | P | 6-Demethoxytangeretin | C19H18O6 | 342.1095 | 9.52 |
| 10 | P | apigenin-7-O-glucuronide | C21H18O11 | 446.084 | 7.2 |
| 11 | P | L-(+)-Arginine | C6H14N4O2 | 174.1115 | 1.02 |
| 12 | P | Synephrine | C9H13NO2 | 167.0944 | 0.92 |
| 13 | P | L-Valine | C5H11NO2 | 117.079 | 0.88 |
| 14 | P | Sinensetin | C20H20O7 | 372.12 | 9.27 |
| 15 | PN | Scopoletin | C10H8O4 | 192.0419 | 5.42 |
| 16 | N | Citric acid | C6H8O7 | 192.0265 | 1.13 |
| 17 | P | 5-O-Methylgenistein | C16H12O5 | 284.0678 | 9.96 |
| 18 | N | Glycyrrhizic acid | C42H62O16 | 822.4046 | 9.04 |
| 19 | N | Corchorifatty acid F | C18H32O5 | 328.2254 | 8.2 |
| 20 | P | Hesperetin | C16H14O6 | 302.0782 | 6.26 |
| 21 | N | Caffeic acid | C9H8O4 | 180.0417 | 3.96 |
| 22 | N | Quinic acid | C7H12O6 | 192.0628 | 0.94 |
| 23 | P | Isosinensetin | C20H20O7 | 372.1198 | 8.77 |
| 24 | N | 3-O-Feruloylquinic acid | C17H20O9 | 368.1111 | 4.65 |
| 25 | N | Cryptochlorogenic acid | C16H18O9 | 354.0956 | 1.75 |
| 26 | P | trans-3-Indoleacrylic acid | C11H9NO2 | 187.0631 | 2.31 |
| 27 | N | Sucrose | C12H22O11 | 342.1167 | 1.28 |
| 28 | N | Baicalein | C15H10O5 | 270.0529 | 6.71 |
| 29 | N | Magnolol | C18H18O2 | 266.131 | 12.57 |
| 30 | N | D-(+)-Malic acid | C4H6O5 | 134.0205 | 0.97 |
| 31 | PN | Hesperidin | C28H34O15 | 610.1878 | 6.27 |
| 32 | P | L-Leucine | C6H13NO2 | 131.0947 | 1.22 |
| 33 | N | Schaftoside | C26H28O14 | 564.1484 | 4.63 |
| 34 | PN | Narirutin | C27H32O14 | 580.1774 | 5.98 |
| 35 | P | Casticin | C19H18O8 | 374.0994 | 9.82 |
| 36 | P | 5,7,3'-Trihydroxy-6,4',5'-trimethoxyflavone | C18H16O8 | 360.0837 | 8.77 |
| 37 | P | Glycyrrhetic acid | C30H46O4 | 470.3382 | 9.5 |
| 38 | N | (15Z)-9,12,13-Trihydroxy-15-octadecenoic acid | C18H34O5 | 330.2409 | 8.67 |
| 39 | N | Limonin | C26H30O8 | 470.1946 | 9.61 |
| 40 | N | Emodin | C15H10O5 | 270.0531 | 11.58 |
| 41 | N | cirsimaritin | C17H14O6 | 314.0795 | 10.21 |
| 42 | N | 2-hydroxycinnamic acid | C9H8O3 | 164.0467 | 5.16 |
| 43 | P | Artemetin | C20H20O8 | 388.1149 | 10.94 |
| 44 | N | Isochlorogenic acid C | C25H24O12 | 516.1273 | 6.17 |
| 45 | N | ADONITOL | C5H12O5 | 152.0677 | 1.04 |
| 46 | P | Genkwanin | C16H12O5 | 284.0676 | 9.5 |
| 47 | N | 7-Demethylsuberosin | C14H14O3 | 230.0942 | 9.63 |
| 48 | PN | eriocitrin | C27H32O15 | 596.1749 | 5.44 |
| 49 | PN | N-acetylphenylalanine | C11H13NO3 | 207.0892 | 5.14 |
| 50 | PN | Kaempferol 3-glucuronide | C21H18O12 | 462.0786 | 5.51 |
| 51 | N | (2R)-2,3-Dihydroxypropanoic acid | C3H6O4 | 106.0253 | 7.84 |
| 52 | N | 4-O-Methylphloracetophenone | C9H10O4 | 182.0573 | 7.82 |
| 53 | N | Syringic Acid | C9H10O5 | 198.0523 | 4.5 |
| 54 | P | Tectoridin | C22H22O11 | 462.1148 | 7.24 |
| 55 | P | Oroxylin A | C16H12O5 | 284.0676 | 6.14 |
| 56 | N | Azelaic acid | C9H16O4 | 188.1043 | 6.37 |
| 57 | N | D-(-)-quinic acid | C7H12O6 | 192.0628 | 0.8 |
| 58 | P | 5-Demethylnobiletin | C20H20O8 | 388.1149 | 8.88 |
| 59 | P | Decursinol | C14H14O4 | 246.0892 | 7.31 |
| 60 | P | Epigoitrin | C5H7NOS | 129.0248 | 2.7 |
| 61 | P | Melibiose | C12H22O11 | 342.1162 | 0.8 |
| 62 | P | 5-Hydroxy-2',4',7,8-Tetramethoxyflavone | C19H18O7 | 358.1046 | 10.48 |
| 63 | P | 3-Epilupeol | C30H50O | 426.3852 | 18.06 |
| 64 | P | Docosanamide | C22H45NO | 339.3491 | 16.42 |
| 65 | P | Pectolinarigenin | C17H14O6 | 314.0784 | 10.22 |
| 66 | N | Dihydroartemisinin | C15H24O5 | 284.1627 | 6.9 |
| 67 | N | Ferulic Acid | C10H10O4 | 194.0573 | 5.53 |
| 68 | N | Methylmalonic acid | C4H6O4 | 118.0258 | 0.98 |
| 69 | PN | Naringenin | C15H12O5 | 272.0679 | 6.16 |
| 70 | N | Phenylacetaldehyde | C8H8O | 120.0564 | 5.16 |
| 71 | N | 2''-O-β-L-Galactopyranosylorientin | C27H30O16 | 610.1545 | 3.46 |
| 72 | P | N-Methylanthranilic Acid | C8H9NO2 | 151.063 | 6.96 |
| 73 | N | Genistein | C15H10O5 | 270.0531 | 9.23 |
| 74 | N | 6-Hydroxycaproic acid | C6H12O3 | 132.0776 | 4.46 |
| 75 | P | 7-Hydroxycoumarin | C9H6O3 | 162.0316 | 8.71 |
| 76 | N | skullcapflavone II | C19H18O8 | 374.1007 | 10.84 |
| 77 | P | Dipteryxin | C17H14O6 | 314.079 | 6.21 |
| 78 | P | 1H-indene-3-carboxamide | C10H9NO | 159.0684 | 0.85 |
| 79 | N | Gluconic acid | C6H12O7 | 196.0577 | 0.79 |
| 80 | PN | Esculin | C15H16O9 | 340.0799 | 2.16 |
| 81 | PN | CHLOROGENIC ACID | C16H18O9 | 354.0956 | 1.51 |
| 82 | N | MUCIC ACID | C6H10O8 | 210.0372 | 1.03 |
| 83 | P | Isotectorigenin, 7-Methyl Ether | C18H16O6 | 328.0941 | 8.2 |
| 84 | P | Guanine | C5H5N5O | 151.0493 | 1.06 |
| 85 | N | Diammonium glycyrrhizinate | C42H62O16 | 822.4049 | 9.95 |
| 86 | N | Dipotassium glycyrrhizinate | C42H60O16 | 820.3881 | 10.49 |
| 87 | P | Iristectorigenin B | C17H14O7 | 330.0732 | 8.61 |
| 88 | N | 4-Methoxysalicylic acid | C8H8O4 | 168.0415 | 1.83 |
| 89 | PN | Apigenin | C15H10O5 | 270.0523 | 8.13 |
| 90 | P | Parthenolide | C15H20O3 | 248.1409 | 9.79 |
| 91 | P | Nobiletin | C21H22O8 | 402.1307 | 10.8 |
| 92 | P | p-Coumaric acid | C9H8O3 | 164.0471 | 5.18 |
| 93 | P | Crotamiton | C13H17NO | 203.1307 | 12.95 |
| 94 | N | 7,8-Dihydroxy-4-methylcoumarin | C10H8O4 | 192.0417 | 6.92 |
| 95 | N | 9(10)-DiHOME | C18H34O4 | 314.2462 | 11.51 |
| 96 | N | PYROCATECHUIC ACID | C7H6O4 | 154.0257 | 4.1 |
| 97 | P | Iridin | C24H26O13 | 522.1358 | 6.26 |
| 98 | N | Daidzein | C15H10O4 | 254.0577 | 7.44 |
| 99 | P | Chrysin | C15H10O4 | 254.0573 | 10.07 |
| 100 | N | 4-Hydroxycoumarin | C9H6O3 | 162.031 | 5.82 |
| 101 | P | Eriodictyol | C15H12O6 | 288.0627 | 5.45 |
| 102 | P | methyl chlorogenate | C17H20O9 | 368.1102 | 4.71 |
| 103 | N | neoeriocitrin | C27H32O15 | 596.175 | 5.27 |
| 104 | N | Gentisic acid | C7H6O4 | 154.0257 | 3.09 |
| 105 | N | Liquiritigenin | C15H12O4 | 256.0737 | 8.91 |
| 106 | P | Isoferulic acid | C10H10O4 | 194.0578 | 8.02 |
| 107 | N | 9-hydroxy-10,12-octadecadienoic acid | C18H32O3 | 296.2354 | 13.06 |
| 108 | N | Stachyose | C24H42O21 | 666.2225 | 0.78 |
| 109 | P | Isovanillin | C8H8O3 | 152.0471 | 5.03 |
| 110 | P | Isofraxidin | C11H10O5 | 222.0524 | 5.58 |
| 111 | N | Isoacteoside | C29H36O15 | 624.2059 | 5.46 |
| 112 | N | Vanillin | C8H8O3 | 152.0464 | 5.02 |
| 113 | PN | Diosmetin | C16H12O6 | 300.0627 | 8.26 |
| 114 | P | Germacrone | C15H22O | 218.1666 | 11.47 |
| 115 | N | Glabrone | C20H16O5 | 336.1003 | 11.58 |
| 116 | P | 5-O-Demethylnobiletin | C20H20O8 | 388.115 | 8.1 |
| 117 | N | Prunin | C21H22O10 | 434.1218 | 6.13 |
| 118 | N | Cyclopentylacetic acid | C7H12O2 | 128.0827 | 5.78 |
| 119 | N | 3-Methylglutaric acid | C6H10O4 | 146.057 | 3.33 |
| 120 | P | Formononetin | C16H12O4 | 268.0729 | 9.06 |
| 121 | PN | Jaceosidin | C17H14O7 | 330.0732 | 8.16 |
| 122 | P | 6-Hydroxypicolinic acid | C6H5NO3 | 139.0268 | 1.02 |
| 123 | P | Oleamide | C18H35NO | 281.2711 | 16.57 |
| 124 | N | p-Hydroxybenzaldehyde | C7H6O2 | 122.0357 | 6.39 |
| 125 | N | ASCORBIC ACID | C6H8O6 | 176.0313 | 7.67 |
| 126 | PN | Hyperoside | C21H20O12 | 464.0962 | 5.37 |
| 127 | P | 6-Hydroxyindole | C8H7NO | 133.0527 | 3.53 |
| 128 | N | 4-Hydroxybenzaldehyde | C7H6O2 | 122.0357 | 5.91 |
| 129 | N | 2-Methylbenzoic acid | C8H8O2 | 136.0514 | 5.03 |
| 130 | P | 3-n-Butylphathlide | C12H14O2 | 190.099 | 10 |
| 131 | N | Nystose | C24H42O21 | 666.2218 | 1.29 |
| 132 | P | Indole-3-acetic acid | C10H9NO2 | 175.0633 | 0.84 |
| 133 | P | alpha-TOCHOPHERYL ACETATE | C31H52O3 | 472.3899 | 16.49 |
| 134 | P | naringenin-7-O-glucoside | C21H22O10 | 434.1202 | 6.15 |
| 135 | P | Sophoricoside | C21H20O10 | 432.1047 | 7.63 |
| 136 | P | osthol | C15H16O3 | 244.1097 | 11.98 |
| 137 | P | Liquiritigenin | C15H12O4 | 256.0733 | 3.65 |
| 138 | N | poliumoside | C35H46O19 | 770.2638 | 5.8 |
| 139 | P | Aurantio-obtusin | C17H14O7 | 330.0731 | 7.93 |
| 140 | P | Homoplantaginin | C22H22O11 | 462.1148 | 4.14 |
| 141 | N | 1-(Carboxymethyl)cyclohexanecarboxylic acid | C9H14O4 | 186.0886 | 7.16 |
| 142 | N | Poncirin | C28H34O14 | 594.1954 | 7.28 |
| 143 | N | 6-Gingerol | C17H26O4 | 294.1835 | 10.48 |
| 144 | P | Didymin | C28H34O14 | 594.193 | 7.29 |
| 145 | P | 8-Hydroxyquinoline | C9H7NO | 145.0525 | 2.39 |
| 146 | P | Xanthotoxol | C11H6O4 | 202.0264 | 8.96 |
| 147 | P | Daidzin | C21H20O9 | 416.1096 | 7.22 |
| 148 | P | 2-(2-amino-3-methylbutanamido)-3-phenylpropanoic acid | C14H20N2O3 | 264.1471 | 3.85 |
| 149 | P | Liquiritin | C21H22O9 | 418.1252 | 5.33 |
| 150 | N | Isosakuranetin | C16H14O5 | 286.0843 | 10.15 |
| 151 | P | cordycepin | C10H13N5O3 | 251.1013 | 1 |
| 152 | P | skimmin | C15H16O8 | 324.0839 | 5.74 |
| 153 | P | Tetramethylpyrazine | C8H12N2 | 136.1 | 1.24 |
| 154 | N | 4-ACETOXYPHENOL | C8H8O3 | 152.0466 | 3.35 |
| 155 | N | IRIGENIN | C18H16O8 | 360.085 | 8.27 |
| 156 | N | 3-Methoxyphenylacetic acid | C9H10O3 | 166.0621 | 6.04 |
| 157 | P | Obacunone | C26H30O7 | 454.1991 | 6.83 |
| 158 | N | tectorigenin | C16H12O6 | 300.0634 | 7.27 |
| 159 | N | norlichexanthone | C14H10O5 | 258.0528 | 8.19 |
| 160 | N | Suberic acid | C8H14O4 | 174.0885 | 5.27 |
| 161 | PN | nicotiflorin | C27H30O15 | 594.1567 | 5.4 |
| 162 | P | 7-Methoxycoumarin | C10H8O3 | 176.0469 | 6.9 |
| 163 | P | cirsimarin | C23H24O11 | 476.1309 | 4.71 |
| 164 | N | 4-Indolecarbaldehyde | C9H7NO | 145.0518 | 6.22 |
| 165 | P | α-Cyperone | C15H22O | 218.1666 | 13.45 |
| 166 | P | Isoliquiritin | C21H22O9 | 418.1251 | 6.7 |
| 167 | N | Luteolin | C15H10O6 | 286.0482 | 6.57 |
| 168 | P | Pectolinarin | C29H34O15 | 622.1876 | 7.17 |
| 169 | P | Melicopidine | C17H15NO5 | 313.095 | 5.95 |
| 170 | P | Vicenin II | C27H30O15 | 594.1572 | 4.54 |
| 171 | N | Epicatechin | C15H14O6 | 290.0795 | 4.16 |
| 172 | N | N-acetyltryptophan | C13H14N2O3 | 246.1005 | 5.66 |
| 173 | P | Fraxetin | C10H8O5 | 208.037 | 3.28 |
| 174 | P | Guanosine | C10H13N5O5 | 283.091 | 0.92 |
| 175 | N | 12-oxo Phytodienoic Acid | C18H28O3 | 292.2041 | 12.01 |
| 176 | N | Mannitol | C6H14O6 | 182.0783 | 0.08 |
| 177 | P | Cholest-4,6-Dien-3-One | C27H42O | 382.3229 | 17.38 |
| 178 | N | 3,3-Dimethylglutaric acid | C7H12O4 | 160.0728 | 4.79 |
| 179 | P | Quillaic acid | C30H46O5 | 486.3328 | 9.04 |
| 180 | PN | RUTOSIDE (rutin) | C27H30O16 | 610.1542 | 5.18 |
| 181 | P | 3,4-di-O-caffeoylquinic acid | C25H24O12 | 516.1257 | 4.44 |
| 182 | N | jaceidin | C18H16O8 | 360.085 | 8.48 |
| 183 | P | Marmesin | C14H14O4 | 246.0887 | 7.74 |
| 184 | N | rhodioloside | C14H20O7 | 300.1212 | 2.01 |
| 185 | P | 4-Pyridineacetic acid | C7H7NO2 | 137.0475 | 2.86 |
| 186 | P | Neohesperidin | C28H34O15 | 610.1879 | 6.61 |
| 187 | P | Isoliquiritigenin | C15H12O4 | 256.0729 | 5.78 |
| 188 | P | Cytosine | C4H5N3O | 111.0433 | 0.88 |
| 189 | P | aloenin | C19H22O10 | 410.1212 | 6.06 |
| 190 | P | Nodakenin | C20H24O9 | 408.141 | 6.29 |
| 191 | N | Uridine | C9H12N2O6 | 244.0698 | 1.1 |
| 192 | P | 7-O-beta-glucopyranosyl-4'-hydroxy-5-methoxyisoflavone | C22H22O10 | 446.12 | 4.67 |
| 193 | P | Alpinetin | C16H14O4 | 270.0884 | 8.55 |
| 194 | P | 3',4',5,7-tetrahydroxyflavone | C15H10O6 | 286.0471 | 7.31 |
| 195 | N | Methyl hexadecanoate | C17H34O2 | 270.2558 | 12.3 |
| 196 | P | Calceolarioside B | C23H26O11 | 478.1464 | 5.74 |
| 197 | N | Lariciresinol 4-O-glucoside | C26H34O11 | 522.2113 | 5.37 |
| 198 | P | Diosmin | C28H32O15 | 608.1725 | 5.89 |
| 199 | PN | Isorhamnetin 3-galactoside | C22H22O12 | 478.11 | 5.75 |
| 200 | N | Dodecanedioic acid | C12H22O4 | 230.1517 | 9.12 |
| 201 | P | 9-Oxo-10(E),12(E)-octadecadienoic acid | C18H30O3 | 294.2187 | 13.74 |
| 202 | P | Oroxylin A-7-O-β-D-glucuronide | C22H20O11 | 460.0991 | 9.59 |
| 203 | P | 4-Aminophenol | C6H7NO | 109.053 | 0.93 |
| 204 | N | homoorientin | C21H20O11 | 448.1008 | 4.69 |
| 205 | P | 2-Hydroxy-4-methoxybenzaldehyde | C8H8O3 | 152.0472 | 2.82 |
| 206 | PN | afzelin | C21H20O10 | 432.1044 | 6.49 |
| 207 | P | Bis(4-ethylbenzylidene)sorbitol | C24H30O6 | 414.2032 | 11.31 |
| 208 | P | Caryophyllene oxide | C15H24O | 220.1824 | 10.45 |
| 209 | P | Vitamin D2 | C28H44O | 396.33864 | 17.88 |
| 210 | P | Artemisinin | C15H22O5 | 282.14631 | 6.77 |
| 211 | N | 2-(Acetylamino)hexanoic acid | C8H15NO3 | 173.1045 | 4.89 |
| 212 | P | Estragole | C10H12O | 148.0888 | 2.32 |
| 213 | P | Kaempferol | C15H10O6 | 286.0469 | 8.28 |
| 214 | P | gerberinside | C16H18O8 | 338.1001 | 5.72 |
| 215 | N | Gallic acid | C7H6O5 | 170.02081 | 1.27 |
| 216 | P | Esculetin | C9H6O4 | 178.02632 | 6.04 |
| 217 | P | Medicarpin | C16H14O4 | 270.08809 | 9.92 |
| 218 | P | apigenin 6,8-digalactoside | C27H30O15 | 594.15715 | 3.89 |
| 219 | P | Carpachromene | C20H16O5 | 336.09911 | 11.57 |
| 220 | P | Poricoic acid A | C31H46O5 | 498.33338 | 13.2 |
| 221 | P | N,N'-Diphenylguanidine | C13H13N3 | 211.1106 | 4.86 |
| 222 | N | 2,5-di-tert-Butylhydroquinone | C14H22O2 | 222.16224 | 10.5 |
| 223 | N | 9-HpODE | C18H32O4 | 312.23 | 13.73 |
| 224 | P | Genistin | C21H20O10 | 432.10471 | 7.12 |
| 225 | P | Apigenin 7-O-(2G-rhamnosyl)gentiobioside | C33H40O19 | 740.21379 | 4.46 |
| 226 | N | 3,5-di-tert-Butyl-4-hydroxybenzyl alcohol | C15H24O2 | 236.17734 | 13.47 |
| 227 | P | Licochalcone A | C21H22O4 | 338.1512 | 11.07 |
| 228 | PN | luteolin 4'-O-glucoside | C21H20O11 | 448.10101 | 5.86 |
| 229 | P | Linderalactone | C15H16O3 | 244.10947 | 9.61 |
| 230 | N | Nervonic acid | C24H46O2 | 366.34929 | 16.75 |
| 231 | P | Glabridin | C20H20O4 | 324.13564 | 11.71 |
| 232 | P | quercetin 3-O-glucuronide | C21H18O13 | 478.07311 | 2.3 |
| 233 | N | L-Histidine | C6H9N3O2 | 155.06848 | 0.78 |
| 234 | P | Astragalin | C21H20O11 | 448.09964 | 5.72 |
| 235 | N | Ethyl caffeate | C11H12O4 | 208.07313 | 7.78 |
| 236 | N | kaempferol 7-O-glucoside | C21H20O11 | 448.10089 | 6.02 |
